# Disruptive *NADSYN1* Variants Implicated in Congenital Vertebral Malformations

**DOI:** 10.3390/genes12101615

**Published:** 2021-10-14

**Authors:** Jiachen Lin, Lina Zhao, Sen Zhao, Shengjie Li, Zhengye Zhao, Zefu Chen, Zhifa Zheng, Jiashen Shao, Yuchen Niu, Xiaoxin Li, Jianguo Terry Zhang, Zhihong Wu, Nan Wu

**Affiliations:** 1 Department of Orthopedic Surgery, Peking Union Medical College Hospital, Peking Union Medical College and Chinese Academy of Medical Sciences, Beijing 100730, China; linjiachen.med@foxmail.com (J.L.); zhaosen830@163.com (S.Z.); zhaozhengye97@163.com (Z.Z.); paragonjeff@163.com (Z.C.); lancetshao@163.com (J.S.); jgzhang_pumch@yahoo.com (J.T.Z.); 2 Beijing Key Laboratory for Genetic Research of Skeletal Deformity, Beijing 100730, China; chivasmo@163.com (Z.Z.); wuzh3000@163.com (Z.W.); 3 Medical Research Center, Peking Union Medical College Hospital, Peking Union Medical College and Chinese Academy of Medical Sciences, Beijing 100730, China; zhaolina19921125@163.com (L.Z.); lsjlab@163.com (S.L.); nhtniuyuchen@126.com (Y.N.); ldx217@yeah.net (X.L.); 4 Key Laboratory of Big Data for Spinal Deformities, Chinese Academy of Medical Sciences, Beijing 100730, China; 5 State Key Laboratory of Complex Severe and Rare Diseases, Peking Union Medical College Hospital, Peking Union Medical College and Chinese Academy of Medical Sciences, Beijing 100730, China

**Keywords:** congenital vertebral malformation, exome sequencing, *NADSYN1* gene, congenital NAD Deficiency Disorder

## Abstract

Genetic perturbations in nicotinamide adenine dinucleotide de novo (NAD) synthesis pathway predispose individuals to congenital birth defects. The *NADSYN1* encodes the final enzyme in the de novo NAD synthesis pathway and, therefore, plays an important role in NAD metabolism and organ embryogenesis. Biallelic mutations in the *NADSYN1* gene have been reported to be causative of congenital organ defects known as VCRL syndrome (Vertebral-Cardiac-Renal-Limb syndrome). Here, we analyzed the genetic variants in *NADSYN1* in an exome-sequenced cohort consisting of patients with congenital vertebral malformations (CVMs). A total number of eight variants in *NADSYN1*, including two truncating variants and six missense variants, were identified in nine unrelated patients. All enrolled patients presented multiple organ defects, with the involvement of either the heart, kidney, limbs, or liver, as well as intraspinal deformities. An in vitro assay using COS-7 cells demonstrated either significantly reduced protein levels or disrupted enzymatic activity of the identified variants. Our findings demonstrated that functional variants in *NADSYN1* were involved in the complex genetic etiology of CVMs and provided further evidence for the causative *NADSYN1* variants in congenital NAD Deficiency Disorder.

## 1. Introduction

Congenital vertebral malformation (CVM) refers to abnormal development of the spine structure presenting as congenital scoliosis, kyphosis, or other congenital vertebral defects and may occur simultaneously with other birth defects or as part of an underlying genetic syndrome [1,2]. In human embryogenesis, the vertebral column develops at 4–6 weeks of gestation from the paraxial mesoderm (PSM) and is closely related to the spinal cord and other organs originating from mesoderm [3,4,5]. Multiple organ defects, most frequently involving the cardiac system, urogenital system, limbs, and spinal cord, have a higher occurrence in CVM than in the general population [3]. A genetic component to CVM risk is suspected. However, only 10~20% of the cases are genetically resolved [6,7,8,9,10].

Nicotinamide adenine dinucleotide (NAD) is biologically essential as a key coenzyme in redox reactions participating in cell metabolism, proliferation and inflammation, as well as circadian rhythm [11,12]. In mammals, NAD is de novo synthesized from L-tryptophan or recycled via the salvage synthesis pathway from endogenous NAD metabolites [12]. Recently, genetic perturbations in the de novo NAD synthesis pathway were identified in individuals with birth defects in vertebral, cardiac, renal organs and limbs, namely the VCRL syndrome [MIM: 617660, 617661, 618845] [13,14]. Truncating variants and disruptive missense variants in the genes encoding key enzymes of the de novo synthesis pathway, including *HAAO*, *KYNU* and *NADSYN1* were identified, respectively. Further investigation revealed that the genetic deletion of the *Haao* or *Kynu* gene, together with deficient dietary NAD precursors during pregnancy, causes VCRL malformations and miscarriages in mice [15]. *NADSYN1*, encoding the final enzyme in the de novo synthesis pathway, NAD synthetase 1, was reported to be a causative gene for congenital NAD Deficiency Disorder [13]. In a previous study, biallelic variants in *NADSYN1* were identified in five individuals from four unrelated families. However, the mutational spectrum of *NADSYN1*-associated congenital disorders has not yet been investigated in a large population cohort.

Here we analyzed the genetic variants in *NADSYN1* in an exome-sequenced cohort consisting of Chinese patients diagnosed with CVM and other congenital organ defects. We further performed in vitro functional assays to investigate the effects of these variants on protein expression and enzyme activity. These findings identified the involvement of functional *NADSYN1* variants in the complex genetic etiology of CVMs.

## 2. Materials and Methods

### 2.1. Patient Recruitment and Clinical Evaluation

A total of 424 probands diagnosed with CVMs were consecutively enrolled and collected in the cohort between 2009 and 2018 at the Department of Orthopedic Surgery of Peking Union Medical College Hospital, as a part of the Deciphering disorders Involving Scoliosis and COmorbidities (DISCO) study (http://www.discostudy.org/ (accessed on 10 January 2019)). Detailed phenotypic data was recorded. X-ray, computed tomography (CT), and magnetic resonance imaging (MRI) were also performed. Deformities of limbs, spine and spinal cord of relevant cases were evaluated via X-ray plain films by two independent surgeons. The cardiac anomalies were evaluated via ultrasonic cardiography. Urogenital and gastrointestinal anomalies were evaluated via ultrasonography of the abdomen. Patients diagnosed with clinical features of VACTERL association (vertebral defects, anal atresia, cardiac defects, tracheo-esophageal fistula, renal anomalies, and limb abnormalities), namely, the anal atresia and tracheo-esophageal fistula were evaluated and ruled out from the study. Physical examination was performed to evaluate the conditions of each patient’s parents. The ethical committee at PUMCH approved the study (IRB number: JS-908). Informed consent was obtained from each participant or their guardians.

### 2.2. Exome Sequencing and Variant Interpretation

Exome sequencing and bioinformatic analysis were conducted. DNA samples were not available for parents. Variants were called, annotated and filtered using the PUMCH developed pipeline (PUMP) as described previously [6]. Rare variants (MAF < 0.001) were selected for analysis based on 1000 Genomes (October 2013), the Exome Aggregation Consortium (ExAC; http://exac.broadinstitute.org (accessed on 10 January 2019)), and the Genome Aggregation Database (gnomAD, http://gnomad.broadinstitute.org/ (accessed on 10 January 2019)). In silico prediction tools, including Sorting Intolerant from Tolerant (SIFT) [16], Polymorphism Phenotyping v2 (Polyphen-2) [17], Genomic Evolutionary Rate Profiling (GERP++) [18] and Combined Annotation Dependent Depletion (CADD) [19] were utilized to predict the deleterious properties of variants. The RefSeq accession numbers of the transcript and corresponding protein isoform of NADSYN1 we used for mutation nomenclature are NM_018161.5 and NP_060631.2, respectively.

### 2.3. Site-Directed Mutagenesis Plasmid Construction

C-terminal Myc-His-tagged NADSYN1 cDNA (NM_018161.5) in pcDNA3.1+ (Hitrobio Biotechnology, Beijing, China) was generated and used as the template for site-directed mutagenesis following the manufacturer’s instructions for the KOD-NEO-PLUS Kit (TOYOBO, Tokyo, Japan). Primers for site-directed mutagenesis at each mutation site were listed in Supplementary Table S1. The mutant plasmids were sequenced on both strands to validate the nucleotide mutation.

### 2.4. Plasmid Transfection and Western Blots

COS-7 cells were acquired from the Institute of Basic Medical Sciences, Chinese Academy of Medical Sciences (Beijing, China). COS-7 cells were cultured in DMEM high glucose (Gibco, Thermo Fisher Scientific, Hudson, NH, USA) supplemented with 10% fetal bovine serum (Gibco, Thermo Fisher Scientific, Hudson, NH, USA) and penicillin-streptomycin solution (50 U/mL, Gibco, USA). COS-7 cells were grown to 60–70% confluency in 6-well plates and then transfected with 2 μg of pcDNA3.1 NADSYN1 WT and all mutant plasmids using Lipofectamine 3000 reagent (Thermo Fisher Scientific, Vilnius, Lithuania). Eight hours after transfection, DMEM high glucose with 20% FBS was added into the transfection mix equivalently. Forty-eight hours post-transfection, after washing with cold PBS, the COS-7 cells were lysed with 80 µL RIPA buffer (Thermo Fisher Scientific, Hudson, NH, USA) containing protease inhibitor cocktail (Roche, Mannheim, Germany).

Then SDS-PAGE and Western blot analysis were conducted on whole cell lysates by standard methods. A rabbit anti-His-tag antibody (1:1000, AE068, ABclonal, Wuhan, China) was used as the primary antibody and goat anti-rabbit horseradish peroxidase-conjugated secondary antibody (ab6721, Abcam, Cambridge, MA, USA) was applied to detect WT and mutant protein expression levels. Then, the cell lysates were re-probed with horseradish peroxidase-conjugated anti-GAPDH antibody (ab9482, Abcam, Cambridge, MA, USA). NADSYN1 and GAPDH expression level were visualized by automatic chemiluminescence imaging system (C300, Azure Biosystems, Dublin, CA, USA). Then quantification of band intensity was performed by ImageJ (National Institutes of Health, Bethesda, MD, USA). Overall expression levels of WT and mutant NADSYN1 were normalized to GAPDH levels, respectively. Statistical comparisons were conducted with the GraphPad Prism software using the one way ANOVA. Each experiment was repeated three times. *p*-Values < 0.05 (*) were considered statistically significant.

### 2.5. Expression and Purification of NADSYN1 Protein in Mammalian COS-7 cells

The COS-7 cells were grown to 60–70% confluency on 2 × 15 cm dishes. Forty-eight hours post-transfection, the cells were lysed on ice for 30 min with PBS + 1%TridonX-100 + 1%NP-40 + 1%PMSF (DMSO free), pH = 7.5, then freeze-thawed 5 times, and ultrasonicated for 10 cycles. WT and mutant NADSYN1 protein was purified using HisPur™ Ni-NTA Purification Kit following the manufacturer’s instructions (88227, Thermo Fisher Scientific, Hudson, NH, USA). The equilibration, wash, and elution buffers were 10 mM, 25 mM, and 500 mM imidazole respectively. The resin beds were incubated with protein extract for 30 min on an end-overend rotor at 4 °C. The eluted proteins were quantified by BCA protein assay kit (Sigma-Aldrich, Darmstadt, Germany).

### 2.6. Enzymatic Assays of NADSYN1 Protein

NADSYN1 protein acts as the final enzyme in NAD^+^ biosynthesis, therefore the content of NAD^+^ was measured using an enzymatic assay described in previous studies [13]. The reaction buffer was prepared by mixing 2 mM ATP, 0.2 mg/mL bovine serum albumin, 5 mM MgCl_2_, 56 mM KCl, 50 mM Tris-HCl (pH 8.0), 20 mM glutamine and 1 mM NaAD. Each reaction system contained 20 µL of reaction mix and 0.2 µg of protein and was then incubated for 60 min at 37 °C and terminated at 95 °C for 5 min. After centrifugation at 13,000 rpm for 15 min, the supernatants were collected for NAD detection. NAD assays were performed in 900 µL of 0.1% ethanol, 10 mM sodium pyrophosphate and 20 units of alcohol dehydrogenase (74931, Sigma-Aldrich, Darmstadt, Germany). The absorbance at 340 nm was measured before and after a 30-min room temperature using Multiskan FC Microplate Photometer (Thermo Fisher Scientific, Hudson, NH, USA). Standard NAD (0–100 nmol) was measured under the same conditions. Statistical analysis of NADSYN1 enzymatic activity were conducted in GraphPad Prism software using one way ANOVA method. Each experiment was repeated three times. *p*-Values < 0.05 (*) were considered statistically significant.

## 3. Results

### 3.1. Identification and Prioritization of NADSYN1 Variants Implicated in DISCO Cohort

Exome sequencing (ES) was performed on 424 sporadic CVM cases from the DISCO study. We identified a total of eight rare *NADSYN1* heterozygous nonsynonymous variants in nine unrelated patients (Table 1). These variants were classified clinically as having unknown functional consequences and, therefore, as VUS in accordance with the American College of Medical Genetics and Genomics (ACMG) guidelines for classification of variant pathogenicity [20]. No pathogenic variants of CVM were identified previously in all of the enrolled patients.

Six missense and two truncating variants were found in 9 unrelated patients. Two of these unrelated patients (SCO2003P0106 and SCO2003P0213) carried the same missense variant (Table 1). Among the six missense variants, three of them were predicted to be deleterious by both SIFT prediction and Polyphen-2 software, with the CADD-PHRED score >20 (c.1037G > A, p.Arg346Gln; c.1762G > A, p.Glu588Lys; c.709G > A, p.Gly237Arg, with the CADD score of 29.6, 20.6, and 23.4, respectively, Table 2). Moreover, the GERP rejected substitution scores of the three variants are >4, suggesting the variants might be functionally disruptive (5.11, 4.83, and 4.64, respectively). Another variant, c.1511G > A(p.Arg504Gln) was also predicted to be deleterious, with a CADD score of 21.1 and a GERP++ score of 3.98. As for the other two missense mutations, c.232G > A(p. Val78Ile) and c.2083G > A(p. Glu695Lys), the in silico prediction was benign/tolerated, with CADD scores of 11.48 and 18.37 and GERP++ scores of 2.99 and 4.35, respectively (Table 2).

### 3.2. Phenotypic Characteristics of Individuals with NADSYN1 Variants

The clinical features of the enrolled individuals are summarized in Table 1. The parents of the enrolled individuals were phenotypically normal based on the physical examination. Vertebral malformations included hemivertebrae, and wedge-shaped vertebrae at the lumbar and thoracic level. Patient SCO2003P0286 presented with preaxial polydactyly. Notably, all of the enrolled patients had extraskeletal abnormalities. Three patients were diagnosed with heart defects, including mitral insufficiency (SCO2003P1980), bicuspid aortic valve (SCO2003P0240), or mitral valve prolapse (SCO2003P0106). Two patients presented renal aplasia (SCO2003P0164 and SCO2003P0286). Four patients were found to have diastematomyelia (SCO2003P0298, SCO2003P1273, SCO2003P0106 and SCO2003P0213), while tethered cords were found among SCO2003P0096 and SCO2003P0213. Additionally, multiple hepatic polycysts were recorded in two of the patients, namely, SCO2003P0298 and SCO2003P0096.

### 3.3. Distribution of the NADSYN1 Variants on the Protein

**Figure 1:**
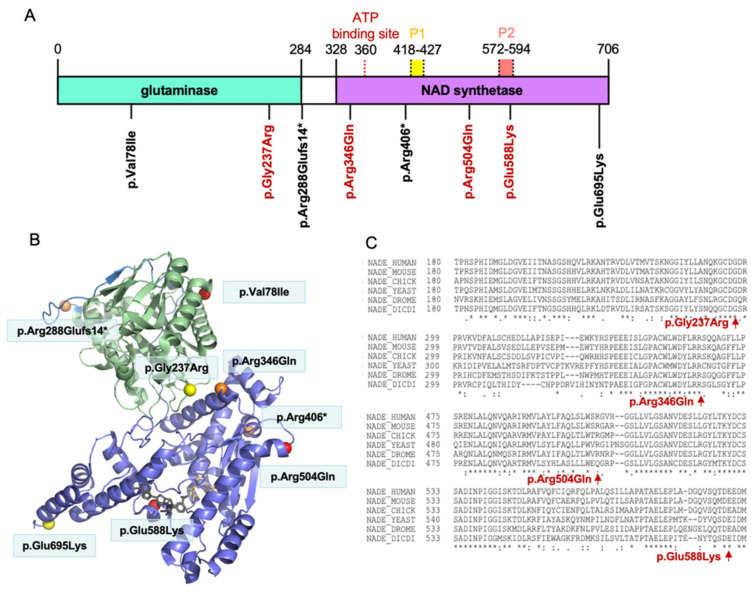
(**A**) Protein domain diagram of human NADSYN1 and the position of identified variants. The glutaminase domain is labeled in green, NAD synthetase domain in purple, P1 loop in yellow, P2 loop in pink, and ATP binding site in red, respectively. The RefSeq transcript sequence of *NADSYN1* gene is NM_018161.5; PDB accession code: 6OFB. (**B**) Space distribution of variants in 3D model using Swiss-Model and PyMOL (https://pymol.org (accessed on 4 January 2021)). (**C**) Position of the variants (red) relative to an evolutionarily conserved region using ClustalW sequence alignment (https://www.genome.jp/tools-bin/clustalw (accessed on 8 April 2021)).

A protein domain diagram of NADSYN1 (Figure 1A) highlights the positions of the identified variants. As demonstrated, the frameshift indel at Arg288 is predicted to cause complete loss of the NAD synthetase domain in the translated protein. Similarly, the stop-gain mutation at Arg406 is predicted to induce abruption of NAD synthetase encoding. The four predictively deleterious variants at Gly237, Arg346, Gln504 and Glu588, highlighted in red, are located in an evolutionarily conserved sequence across species (Figure 1C). Notably, the variant p.Glu588Lys presents within the conserved P2 loop of NAD synthetase, similar to the previously reported functional variant p.Ala573Thr in VCRL patients [13]. Additionally, p.Arg504Gln is also located next to the P2 loop site. In contrast, the variant p.Arg346Gln surrounds the ATP-binding site at amino acid Val360 [21]. We then visualized the space distribution of variants in the 3D model using the Swiss-Model and PyMOL (PDB ID: 6OFB, https://pymol.org (accessed on 4 January 2021)) [22] (Figure 1B). The variant p.Gly237Arg, as demonstrated in the glutaminase domain, is found to be close to the ATP-binding domain the same as variant p.Arg346Gln. These findings suggested that the above missense variants located closely to the critical functional domains of NADSYN1 protein.

### 3.4. Functional Assessment of the NADSYN1 Variants

**Figure 2:**
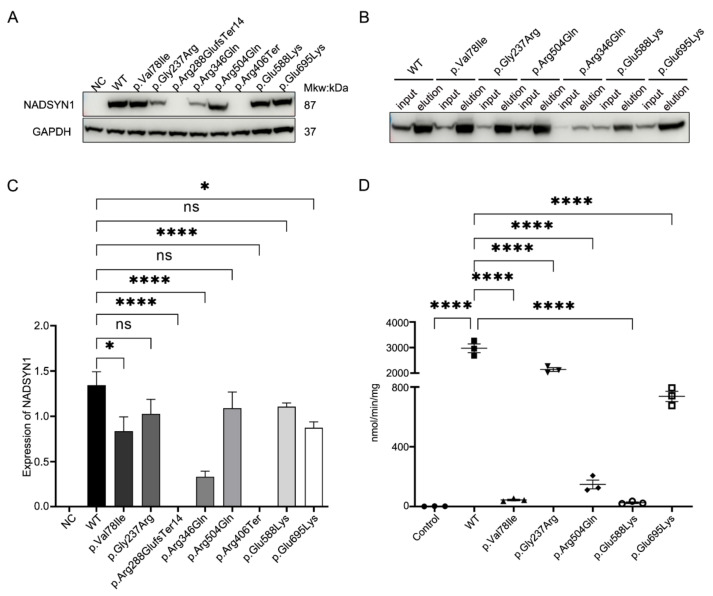
(**A**) Expression of NADSYN1 protein in cells transfected with wild type (WT) and mutant NADSYN1 plasmid. (**B**) The western blot shows the content of the NADSYN1 protein before and after protein purification. (**C**)Quantification of the NADSYN1 WT and mutant protein based on western blot results. Then band intensity was measured by ImageJ based on ImageJ software. (**D**) Nicotinamide adenine dinucleotide synthetase activity of purified mutant protein compared to wild-type (WT) NADSYN1 protein. Abbreviations: NC, negative control; ns, non-significant. The results are shown as the mean ± standard error of three independent experiments. *p*-Values < 0.05 (*), 0.0001 (****) were considered significant.

To test the effect of all the *NADSYN1* variants on protein function, we overexpressed either human *NADSYN1* wild-type or mutant protein in COS-7 cells. Forty-eight hours after transfection, total cell lysate was collected and plasma protein extracted. We then performed SDS-PAGE and Western blot analysis on the whole cell lysate from WT and the respective mutants (Figure 2A,C). The results demonstrated a low expression level of endogenous NADSYN1 protein in COS-7 cell line. A loss of expression of complete protein after transfecting the truncating variants, c.861delT(p.Arg288GlufsTer14) and c.1216C > T(p.Arg406Ter) was noted (Figure 2A, both *p* < 0.001), possibly due to the non-sense mediated decay (NMD) effects in the cells. Compared to wild-type, significantly decreased expression of variant c.232G > A(p.Val78Ile) (*p* < 0.05), c.1037G > A(p.Arg346Gln) (*p* < 0.001), and c.2083G > A(p.Glu695Lys) (*p* < 0.05) were observed, suggesting a potentially decreased protein stability caused by the variants. The other three variants, c.709G > A(p.Gly237Arg), c.1511G > A(p.Arg504Gln), and c.1762G > A(p.Glu588Lys) did not show altered protein expression levels as compared to that of the wild type.

Previous studies have demonstrated that deleterious *NADSYN1* variants could generate NAD synthetase with impaired enzymatic activity [13]. We further tested the enzymatic activities of wild-type and mutants using a previously described method [13]. We were not able to generate enough purified NADSYN1 from the variant c.1037G > A(p.Arg346Gln) due to the low protein expression. The enzymatic activities of NAD of wild-type and five other mutants were assessed (Figure 2D). We found a significantly decreased level of mutant enzymatic activity. The mutant c.232G > A(p.Val78Ile) and c.1762G > A(p.Glu588Lys) demonstrated a 100–150 fold decrease compared to wild-type (both *p* < 0.001), and the c.1511G > A(p.Arg504Gln) demonstrated an ~20-fold decrease in enzymatic activities (*p* < 0.001). Additionally, c.2083G > A(p.Glu695Lys) demonstrated an ~3.75-fold and c.709G > A(p.Gly237Arg) an ~1.5-fold decrease in enzymatic activity (both *p* < 0.001). Notably, both variants on the glutaminase domain (c.232G > A, c.709G > A) and NAD synthetase domain (c.1511G > A, c.1762G > A and c.2083G > A) (Figure 1) resulted in a significantly decreased NAD synthesis ability.

## 4. Discussion

In the present study, we identified eight potentially disruptive variants in a total number of nine CVM patients, with subsequent functional assessment. All patients presented with CVMs and other defects, either in heart, kidney, limbs, or spinal cord. We further evaluated the pathogenicity of these variants using in silico prediction methods, and tested the protein expression level and enzymatic activity in the COS-7 cell line. These findings provided further evidence of the involvement of NAD deficiency in congenital organ defects.

Nicotinamide adenine dinucleotide (NAD) is essential for mammals and serves endogenously as a key coenzyme in redox reactions and a precursor for cell metabolism, and also a substrate for protein modifications [11,12]. Congenital NAD deficiency due to genetic perturbations and dietary deficiency of NAD precursors would impair organogenesis both in humans and mice [14,15]. It was reported that genetic loss of non-redundant de novo NAD synthesis genes, *KYNU* and *HAAO*, was associated with the VCRL syndrome, which is characterized by congenital vertebral malformations and other organ defects. The vertebral phenotype of humans was reproduced in mouse models with genetic deletion of *Kynu* and *Haao*, complicated with dietary deficiency of NAD precursors in pregnant mice. These data suggested that congenital NAD Deficiency Disorder may be caused by a gene–environment interaction.

*NADSYN1* encodes the final enzyme of the NAD de novo synthesis pathway [23]. The role of *NADSYN1* during organ development has not yet been elucidated. Notably, all five individuals presented in a previous study did not survive more than three months postnatally [13]. *NADSYN1*, like many non-redundant genes in the NAD synthesis pathway, appears tolerant to heterozygous missense mutations in humans. Here, we present nine individuals carrying heterozygous deleterious variants of *NADSYN1* with multiple organ defects who survived postnatally with relatively good quality of life (admitted to the hospital from age 1 to 22 years). In addition to CVM, all patients in this study presented with other organ defects involving either the heart, limbs, kidney, liver or spinal cord. Notably, two of the patients presented with multiple hepatic polycysts, which has not been previously reported in NAD Deficiency Disorder cases. Considering that congenital NAD deficiency disorder is caused by a gene–environment interaction, we propose that functional *NADSYN1* variants are involved in CVMs with variable penetrance and expressivity.

The functional NADSYN1 protein contains glutaminase and synthetase domains [21] and catalyzes the ATP-dependent formation of NAD+ from nicotinic acid adenine dinucleotide (NaAD+) at the synthetase domain using the ammonia generated from the glutaminase domain [23,24]. It has been previously reported that variants in both the glutaminase (p. Cys49Arg) domain and synthetase domain (p. Ala573Thr) will cause a significant decrease in NAD synthesis activities of encoded *NADSYN1* [13]. To validate the proposed hypothesis, we performed in vitro assays to evaluate the consequences of *NADSYN1* variants located in different domains. One frameshift variant, c.861delT(p. Arg288GlufsTer14), led to complete loss of the NAD synthetase domain in the translated protein (Figure 2A,B). Another possible explanation is that the non-sense mediated decay (NMD) is activated in COS-7 cells to mediate the degeneration of mutant mRNA transcripts from variant c.861delT(p. Arg288GlufsTer14) and c.1216C > T(p.Arg406Ter). Two missense variants in the NAD synthetase domain, c.1511G > A(p. Arg504Gln) and c.1762G > A(p. Glu588Lys), are structurally close to the P2 loop, the active site for NAD synthetase [21], significantly decreasing the enzymatic activity (Figure 1A). Consistent with previous results, the missense variant in the glutaminase domain, c.232G > A(p. Val78Ile), demonstrated significantly decreased enzymatic activity [13]. However, given the fact that congenital Deficiency Disorder is known to be caused by biallelic variants in *NADSYN1*, we still consider these variants as uncertain significance.

As a multifactorial disease with a prevalence of ~1/1000 live births [1,7], genetic perturbations, gene–environment interactions, and epigenetic changes are suspected to play a role in the etiology of CVMs [4,8]. Previously, we have reported the mutational burden and potential oligogenic models underpinning CVM development, as well as compound heterozygous inheritance model in *TBX6*-associated congenital scoliosis (TACS) [9,25,26]. The combined effect of deleterious variants in multiple genes might synergistically lead to the development of the malformations, which gives insight into the complex disease-causing model of CVMs. Apart from genetic perturbations, hypoxia, insufficient folate acid supply, and teratogenic drug use during pregnancy are also considered to be potential CVM risk factors [8]. Considering the complexity of NAD-involved biological processes, the exact mechanism underpinning NAD-mediated organogenesis defects remains to be elucidated. As anticipated, defects in organs originating from the mesoderm in the cardiac system and urogenital system, as well as limbs, often co-occur with somitogenesis defects in other congenital disorders [3,5,27], highlighting the vital role of *NADSYN1* during embryogenesis of the mesoderm.

In summary, functional variants in *NADSYN1* were involved in the complex genetic etiology of CVMs. Further investigation of congenital NAD Deficiency Disorder will help to understand the pathogenesis of syndromic congenital defects involving CVMs.

## Figures and Tables

**Table 1:** Summary of clinical features of the *NADSYN1* variant carriers from the DISCO cohort. Patients were diagnosed with congenital scoliosis presenting with vertebral deformities and other malformations. The RefSeq transcript sequence of the *NADSYN1* gene is NM_018161.5. Abbreviations: Het, heterozygous; M, male; F, female; yo, years-old; T, thoracic vertebra; L, lumbar vertebra.

Patient Number	Variant	Zygosity	Sex	Age at Admission	Vertebral Deformities	Other Malformations
SCO2003P0298	c.232G > A(p.Val78Ile)	Het	F	12 yo	T3, T5, T6, T7, T8 vertebral malformation and unsegmentation, T10–T11 unsegmentation	Diastematomyelia, multiple hepatic polycysts, maxillary sinusitis
SCO2003P1273	c.709G > A(p.Gly237Arg)	Het	M	13 yo	T2–L3 vertebral malformation and unsegmentation	Diastematomyelia, spina bifida, mucocele
SCO2003P0164	c.861delT(p.Arg288GlufsTer14)	Het	M	11 yo	T6 butterfly vertebra, T7 hemivertebra, T5–T9 fused vertebral plate; T7 rib absent, T9–T10 fused rib	Renal aplasia (left)
SCO2003P0240	c.1037G > A(p.Arg346Gln)	Het	M	6 yo	T10, T11, T12 hemivertebrae	Bicuspid aortic valve
SCO2003P1980	c.1216C > T(p.Arg406Ter)	Het	F	1 yo	T6–T10 unsegmentation	Mitral insufficiency
SCO2003P0096	c.1511G > A(p.Arg504Gln)	Het	F	9 yo	L2 wedge-shape vertebra, L3 wedge-shape vertebra, L1–L4 unsegmentation	Tethered cord, multiple hepatic polycysts, splenomegaly
SCO2003P0286	c.1762G > A(p.Glu588Lys)	Het	F	1 yo	T3–T8 vertebral malformation and unsegmentation	Renal aplasia (left), polydactyly (left), dilatation of central canal of spinal cord
SCO2003P0106	c.2083G > A(p.Glu695Lys)	Het	M	10 yo	T7, T8, T9 butterfly vertebrae, T6–T10 unsegmentation	Diastematomyelia, mitral valve prolapse
SCO2003P0213	c.2083G > A(p.Glu695Lys)	Het	M	22 yo	T8–T9 unsegmentation, T9–T10 rib bifurcation	Diastematomyelia, tethered cord

**Table 2:** Sequence variants in *NADSYN1* and their statistics in gnomAD statistics, in silico prediction scores, and in vitro assay results. The RefSeq transcript sequence of the *NADSYN1* gene is NM_018161.5. Abbreviations: NA, not available.

Patient Number	cDNA Change	Protein Change	gnomAD * Allele Frequency	SIFT Prediction Score	Polyphen-2	GERP++	CADD-PHRED Score
SCO2003P0298	232G > A	Val78Ile	0	0.5	0.013	4.35	18.37
SCO2003P1273	709G > A	Gly237Arg	0	0	0.974	4.64	23.4
SCO2003P0164	861del	Arg288GlufsTer14	0	NA	NA	NA	NA
SCO2003P0240	1037G > A	Arg346Gln	0	0.01	0.994	5.11	29.6
SCO2003P1980	1216C > T	Arg406Ter	0.0000083	NA	NA	3.05	42
SCO2003P0096	1511G > A	Arg504Gln	0.00013	0.06	0.378	3.98	21.1
SCO2003P0286	1762G > A	Glu588Lys	0	0	1	4.83	20.6
SCO2003P0106/SCO2003P0213	2083G > A	Glu695Lys	0.00022	0.21	0.01	2.99	11.48

* Dataset based on highest MAF in population.

## Data Availability

The datasets used and/or analyzed during the current study are available from the corresponding authors upon reasonable request.

## Supplementary Materials

The following are available online at https://www.mdpi.com/article/10.3390/genes12101615/s1. The primers for site-directed mutagenesis of the NADSYN1 plasmids are summarized in Supplementary Table S1.
