# Supplementary material for: Disruptive NADSYN1 Variants Implicated in Congenital Vertebral Malformations
Source: Genes (Basel). 2021 Oct 14;12(10):1615. doi: 10.3390/genes12101615 (PMC8535205; doi:10.3390/genes12101615)
Supplement: Supplementary file 1 [file genes-12-01615-s001.zip › Supplementary Table 1 - updated.pdf]

Supplementary Table S1. Primers for site-directed mutagenesis of NADSYN1 gene mutants.

Primers	5’-3’
NADSYN1-C1216T-F	ATCCCtGAGACCTCTGTGGACGCATACTGACC
NADSYN1-C1216T-R	ACAGAGGTCTCaGGGATCCTGGGGGGTGTAGC
NADSYN1-861delT-F	TTTCATCCGAAACCTGGCGGCCAGCAGGGCGAG
NADSYN1-861delT-R	GCCAGGTTTCGGATGAAATCTCCGCCCTGTAGC
NADSYN1-G1037A-F	AGACaAAGTCAACAGGCAGGGTTTTTGCTGCC
NADSYN1-G1037A-R	GCCTGTTGACTTtGTCTTAAAAAATCCCAGAGCCAGC
NADSYN1-G1762A-F	AGACCGACaAGGAAGATATGGGGATGACATATGC
NADSYN1-G1762A-R	ATCTTCCTtGTCGGTCTGGGACACCTGTCCAT
NADSYN1-G1511A-F	TTGAGCCTCTGGTCTCaGGGTGTCCACGGTGGGCT
NADSYN1-G1511A-R	tGAGACCAGAGGCTCAACTGAGCAAACAGATA
NADSYN1-G2083A-F	TACAGCTCaAGAGGGCAGAGCCACAGTCCCTG
NADSYN1-G2083A-R	TGCCCTCTtGAGCTGTAGCACCTGATTTTCTATGC
NADSYN1-C2014T-F	GGTTTGATCTGtGACCATTTCTGTACAACACAAGCTG
NADSYN1-C2014T-R	TGGTCaCAGATCAAACCTGTTGTCCTCAGGGC
NADSYN1-G232A-F	AGTCTCCCaTCACTCAGGACATCATCTGCGAC
NADSYN1-G232A-R	CTGAGTGAtGGGAGACTCCACAAGGGCCGCTA
NADSYN1-G709A-F	AAGGGTTGTGACaGGGACCGCCTGTACTACGACG
NADSYN1-G709A-R	TCCCtGTCACAACCCTTCTGGTTGGCCAGCAA
